# Understanding the dynamic interactions of root-knot nematodes and their host: role of plant growth promoting bacteria and abiotic factors

**DOI:** 10.3389/fpls.2024.1377453

**Published:** 2024-04-30

**Authors:** Alemayehu Habteweld, Mihail Kantor, Camelia Kantor, Zafar Handoo

**Affiliations:** ^1^ Mycology and Nematology Genetic Diversity and Biology Laboratory, USDA, ARS, Northeast Area, Beltsville, MD, United States; ^2^ Plant Pathology and Environmental Microbiology Department, Pennsylvania State University, University Park, PA, United States; ^3^ Huck Institutes of the Life Sciences, Pennsylvania State University, State College, PA, United States

**Keywords:** root-knot nematodes, root-knot nematode-host interactions, plant growth promoting bacteria, root exudates, volatiles, biotic factors, abiotic factors, agricultural practices

## Abstract

Root-knot nematodes (*Meloidogyne* spp., RKN) are among the most destructive endoparasitic nematodes worldwide, often leading to a reduction of crop growth and yield. Insights into the dynamics of host-RKN interactions, especially in varied biotic and abiotic environments, could be pivotal in devising novel RKN mitigation measures. Plant growth-promoting bacteria (PGPB) involves different plant growth-enhancing activities such as biofertilization, pathogen suppression, and induction of systemic resistance. We summarized the up-to-date knowledge on the role of PGPB and abiotic factors such as soil pH, texture, structure, moisture, etc. in modulating RKN-host interactions. RKN are directly or indirectly affected by different PGPB, abiotic factors interplay in the interactions, and host responses to RKN infection. We highlighted the tripartite (host-RKN-PGPB) phenomenon with respect to (i) PGPB direct and indirect effect on RKN-host interactions; (ii) host influence in the selection and enrichment of PGPB in the rhizosphere; (iii) how soil microbes enhance RKN parasitism; (iv) influence of host in RKN-PGPB interactions, and (v) the role of abiotic factors in modulating the tripartite interactions. Furthermore, we discussed how different agricultural practices alter the interactions. Finally, we emphasized the importance of incorporating the knowledge of tripartite interactions in the integrated RKN management strategies.

## 1 Introduction

Plant-parasitic nematodes (PPNs) infect a wide range of food crops and cause severe damage (Nicol et al., 2011; Jones et al., 2013; Kantor et al., 2022). PPNs control costs several billions of dollars annually to the global agriculture industry (Elling, 2013; Gamalero and Glick, 2020; Kantor et al., 2022). Among these, root-knot nematodes (RKN) are the most economically significant plant pathogens due to the high levels of damage and infection they cause, their wide host and geographic ranges, and interaction with other plant pathogens (Rehman et al., 2012; Topalović and Geisen, 2023). The second-stage juvenile (J2) is the only infective stage of RKN. In plant roots, the J2s undergo two developmental stages (J3 and J4) before an adult stage. Adult females establish feeding sites and cause root galls (Eisenback and Triantaphyllou, 1991; Karssen and Moens, 2006; Gheysen and Mitchum, 2011).

The above-ground symptoms of RKN-infected plants include poor plant growth, necrosis on leaves, and rapid wilting under environmental stress caused by water deficiency or other factors (Bernard et al., 2017; Elnahal et al., 2022). The obvious below-ground symptom of RKN infection is the formation of galls on the roots that reduce the absorption and translocation of water and dissolved nutrients. RKN root damage also fosters access to secondary infection of roots by soil pathogens such as fungi and bacteria (Agrios, 2005; Cao et al., 2023). Because of their wide host range and distribution, effective RKN management is becoming a global priority. Effective RKN management may require an integrated application of control strategies such as chemical nematicides, resistant crops, trap crops, organic amendments, and different microbial agents (Desaeger et al., 2020; Forghani and Hajihassani, 2020; Topalović et al., 2020b). Synthetic chemical nematicides are effective in controlling RKN and are widely used around the globe but their use has been restricted due to their negative impact on human health and the environment (Katooli et al., 2010; Desaeger et al., 2020). Thus, there is a critical need for alternative nematode control methods which are both effective in controlling RKN and environmentally sustainable.

RKN management strategies using antagonistic soil microbiota would offer an ecologically sound RKN control (Zhang et al., 2017; Abd-Elgawad, 2021; Aioub et al., 2022). A broad range of soil microbiota reduced nematode infection directly or indirectly in plants (Eberlein et al., 2016; Ashrafi et al., 2017; Hamid et al., 2017; Hussain et al., 2018; Nuaima et al., 2021). These microbes use antibiosis, parasitism, induced systemic resistance (ISR) in plants, or apply a combination of different strategies that can interfere with nematode infection in plants (Chen and Dickson, 1998; Siddiqui et al., 2005; Martínez-Medina et al., 2017; Poveda et al., 2020). One subset of soil microbiota showing RKN suppression is plant growth promoting bacteria (PGPB). Here, we define PGPB as bacterial community inhabiting soil around roots (rhizosphere bacteria) and inside plant roots (endophytic bacteria) and promoting plant growth through a variety of processes such as biofertilization, phytohormone production, antipathogenic activities and ISR (Lugtenburg and Kamilova, 2009; Aioub et al., 2022; Gowda et al., 2022).

PGPB stimulate plant growth by supplying essential plant nutrients such as nitrogen (N), phosphorus (P), potassium (K), and other micronutrients such as iron (Fe), and promote soil bioremediation by secreting a variety of metabolites and hormones (Poria et al., 2022). PGPB have a direct influence on both plant development and metabolism through the production of phytohormones and plant growth regulators (Chandra et al., 2018). Microbial phytohormones and phytostimulators such as auxins, ethylene, cytokines, gibberellin, abscisic acid, salicylic acid and jasmonic acid are important in plant biological processes such as cell division and elongation (Lugtenburg and Kamilova, 2009; Tsukanova et al., 2017; Shaffique et al., 2023). Phytohormones and enzymes such as 1-aminocyclopropane-1-carboxylate (ACC) produced by PGPB also alleviate various forms of stress, including infections by pathogenic bacteria, resistance to stress induced by polyaromatic hydrocarbons, heavy metals such as Ni^2+^ and environmental stresses such as salt and drought (Glick et al., 2007; Lugtenburg and Kamilova, 2009; Zhang et al., 2020; Sharifi et al., 2022). PGPB are also involved in antipathogenic activities through antagonism, signal interference, predation, parasitism, competition, and induced systemic resistance (ISR) (Milner et al., 1996; Bassler, 1999; Ryu et al., 2003; Emmert et al., 2004; Aioub et al., 2022). Thus, the use of PGPB for RKN control is an ecologically sound strategy for suppressing RKN using naturally occurring species, introducing them to the rhizosphere or manipulating the soil through different agricultural practices that enhance their performance (Topalović et al., 2019; Topalović et al., 2020b; Eldeeb et al., 2022).

**Figure 1:**
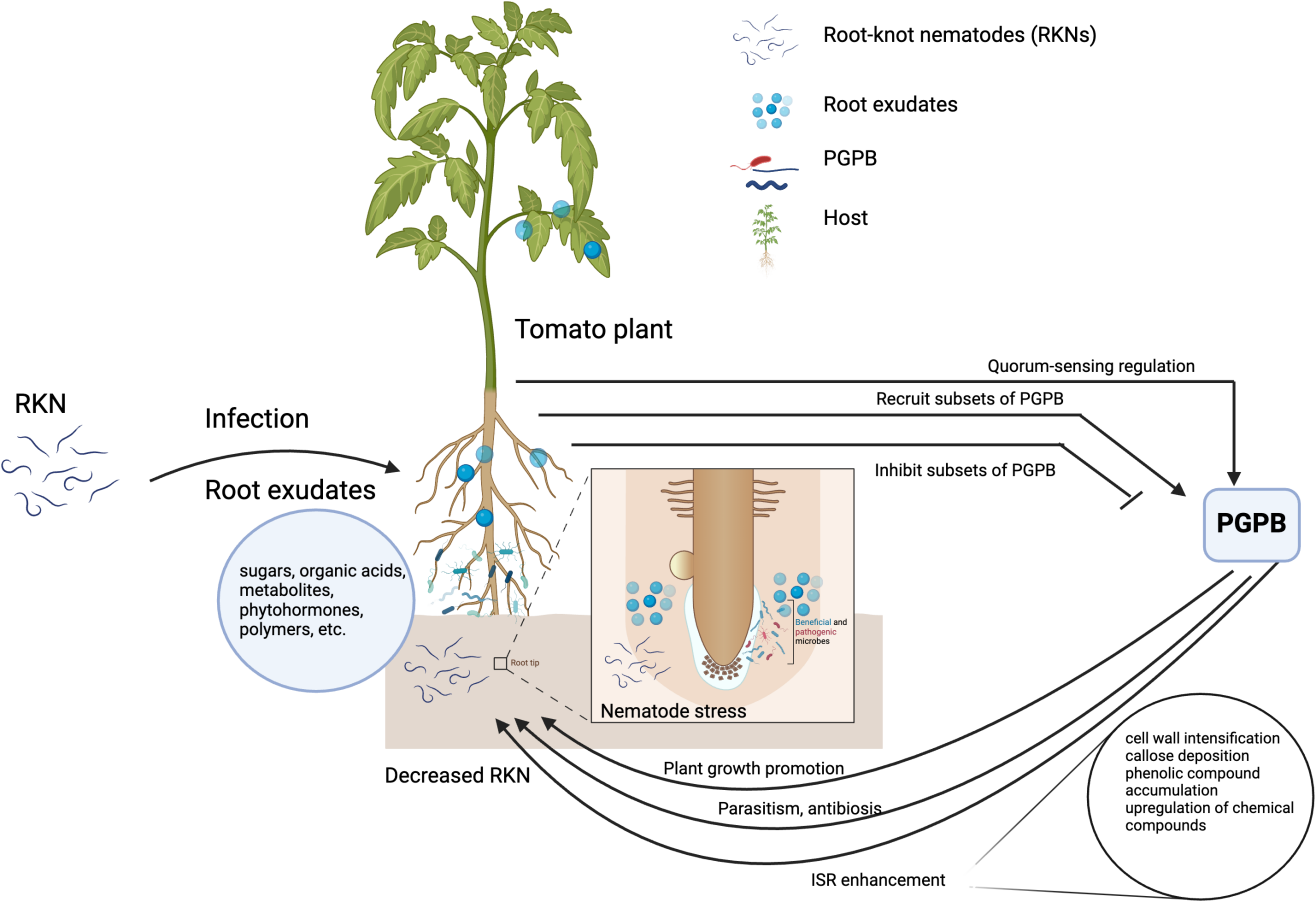
Simplified diagrammatic representation of host-RKN-PGPB interactions. Host plants shape the rhizosphere microorganisms by recruiting or inhibiting subsets of plant growth promoting bacteria (PGPB) for its benefits. The PGPB directly attack the root-knot nematodes and/or through plant growth promotion and induced systemic resistance. The figure is created with BioRender.com.

Despite several promising RKN control results under laboratory and greenhouse settings, transitioning from lab to field has been challenging due to inconsistencies in microbial agent performance in the field conditions (Topalović and Heuer, 2019). Some reasons for that are the weak competitive ability of the microbial agents and the failure to establish a high density in soil ecosystems. In soil ecosystems, host-RKN interactions are complex and can be affected by different soil physicochemical and biological properties (Cao et al., 2023). Soil physicochemical and biological properties modulate host-RKN interactions, and they are in turn affected by different agricultural practices. Therefore, the knowledge of how soil biotic and abiotic factors modulate the host-RKN-PGPB (tripartite, **Figure 1**) interactions would be critical to develop economically and ecologically sound RKN control strategies. This review emphasizes the tripartite phenomenon with respect to (i) PGPBs’ direct and indirect effect on RKN-host interactions; (ii) the host’s influence in the selection and enrichment of PGPB in the rhizosphere; (iii) the influence of the host in RKN-PGPB interactions; (iv) the role of soil microbes in enhancing RKN parasitism, and (v) the role of abiotic factors in modulating the tripartite interactions. Furthermore, the paper delves into how different agricultural practices alter the host-RKN-PGPB interactions. Finally, it concludes by emphasizing the importance of incorporating the knowledge of tripartite interactions in the integrated RKN management strategies.

## 2 Influence of PGPB on host-RKN interactions

PGPB have a direct and indirect effect on RKN and affect the interaction between the host and RKN (**Figure 1**).

### 2.1 Direct modulatory mechanisms

Direct modulatory mechanisms refer to the adverse effect of PGPB on RKN through processes such as parasitism and antibiosis. Among these, parasitism stands out as the most effective and direct modulatory mechanism which entails the tropic growth of the PGPB toward RKN, ultimate assault, and disintegration of RKN through enzyme activity (Pal and Gardener, 2006; Singh et al., 2017). For instance, *Pasteuria penetrans* showed strong parasitism to *Meloidogyne graminicola*, *M. incognita*, *M. arenaria*, and *M. hapla* (Ciancio, 2018; Aioub et al., 2022).

Antibiosis involves the release of enzymes, metabolic by-products, and toxins by various PGPB to actively suppress RKNs. The release of these products plays a crucial role in inhibiting nematode hatching, development, and existence (Subedi et al., 2020). Some PGPB such as *Pseudomonas fluorescens*, produce metabolic by-products like 2,4-diacetylphloroglucinol and hydrogen cyanide that suppress RKN density and promote plant growth (Mena et al., 2002; Meyer et al., 2009; El-Rahman et al., 2019). Research on *Corynebacterium paurometabolous* and *Lysobacter capsica* showed that these PGPB expressed chitinase and gelatinase activities which reduced the numbers of galls and egg masses of *M. incognita* in tomatoes (Mena et al., 2002; Lee et al., 2015). *Bacillus cereus* strain (BCM2) released 2,4-2,4-ditert-butylphenol, 3,3 dimethyloctane, chitonase, alkaline serine protease, and neutral protease which resulted in the reduction of *M. incognita* density on tomato (Li et al., 2019a). *B*. *thuringiensis* is known to produce proteinaceous protoxin crystals that cause lysis of the intestine and the nematode’s death (Griffitts et al., 2005; Vachon et al., 2012; Elhady et al., 2017).

Several PGPB strains with antibiosis activity such as *Bacillus aryabhattai* A08, *Paenibacillus alvei* T30, *Bacillus firmus* T11, *Paenibacillus barcinonensis* A10, and *B. cereus* N10w were reported (Viljoen et al., 2019). Application of *P. fluorescens* and *Serratia marcescens* reduced the number of galls and egg masses of RKN species (Ali et al., 2021). *Pseudomonas* spp. and *Bacillus* spp. isolated from RKN suppressive soil exhibited RKN antagonism on tomato (Zhou et al., 2019). Similarly, soils with low- and high-infestations of *M. incognita* demonstrated different microbial communities in the rhizosphere, the low-infested soil contained more of plant beneficial microbes including those with nematocidal activities such as *B. amyloliquefacens* W1 (Zhao et al., 2023).

### 2.2 Indirect interventional mechanisms

PGPB indirectly suppress RKN by promoting plant growth and development through improved nutrient availability and uptake, producing phytohormones, and activating ISR. Recent studies revealed that N-fixing PGPB strains promote plant growth by providing nitrogen and increase plants’ resistance to RKN (Aioub et al., 2022). For instance, *Rhizobium* spp., *Mesorhizobium* spp., *Sinorhizobium* spp., *Bradyrhizobium* spp. and *Frankia* spp. fix N_2_, supply it to plants and promote plant growth (Vejan et al., 2016; Dash et al., 2017; Liao et al., 2021). Similarly, *Paenibacillus polymyxa*, a N-fixing PGPB, promoted plant growth and suppressed *Meloidogyne incognita* populations on tomato in a greenhouse experiment (El-Hadad et al., 2011). *Azospirillum* spp., *Azotobacter* spp., and *Rhizobium* spp. decreased root galling caused by *Meloidogyne javanica* in root of chickpea (*Cicer arietinum*) (Siddiqui and Mahmood, 2001). Inoculating tomato plants with *Bacillus firmus*, *B. megaterium* and *B. circulans* also improved plant growth through phosphate acquisition and suppressed *M. incognita* populations (Terefe et al., 2009; El-Hadad et al., 2011). Likewise, phosphate-solubilizing *P. fluorescens* suppressed *M. incognita* population in chickpea field (Rizvi et al., 2012). PGPB with phytohormone-producing ability promoted plant growth and suppressed PPNs (Backer et al., 2018). For instance, indole acetic acid (IAA) production by the strain *Streptomyces fradiae* NKZ-259 enhanced plant growth (Myo et al., 2019). *Pseudomonas simiae* strain MB751 also produced IAA, improved plant growth, and suppressed *M. incognita* development (Sun et al., 2021).

PGPB decrease the population of RKN by enhancing ISR through eliciting plant innate immunity in plants. This is accomplished through processes like cell wall intensification, callose deposition, phenolic compound accumulation, and upregulation of biochemical compounds such as jasmonic acid, pathogenesis-related proteins, lipopolysaccharides, phytoalexin, siderophores, chitinase, and salicylic acid (Aioub et al., 2022). For instance, *M. javanica* and *M. incognita* population densities were suppressed because of the activation of ISR when *Arabidopsis* roots were treated with PGPB *Bacillus cereus* (Jiang et al., 2020). Similarly, tomato roots inoculated with *P. fluorescens* Pf128 and *B. subtilis* Bbv57 decreased *M. incognita* populations due to increased activity of enzymes involved in ISR (Meena et al., 2012).

## 3 Plants recruit and shape PGPB communities in the rhizosphere

Different plant species selectively attract different communities of PGPB and influence their composition when grown on the same soil (Berendsen et al., 2012). The PGPB exhibit significantly higher population densities in the rhizosphere compared to the bulk soil, primarily due to plants releasing up to 40% of their photosynthates as root exudates (Bais et al., 2006). However, their diversity is low in the rhizosphere compared to the bulk soil (Berg et al., 2006; Costa et al., 2006; Hein et al., 2008; Berendsen et al., 2012) indicating PGPB community establishment is driven by host plant selection (Li et al., 2019b; Yin et al., 2021). The type and age of the host plant, and biotic and abiotic stresses influence the compositions of root exudates (Lundberg et al., 2012; Chaparro et al., 2014; Bulgarelli et al., 2015; Tkacz et al., 2015; Kantor et al., 2018; Yin et al., 2021). Thus, the composition of root exudates actively secreted by plants shape the PGPB community by stimulating or repressing the subset of the PGPB community in the soil (Doornbos et al., 2012). Components of root exudates such as sugars, organic acids, metabolites, phytohormones, and complex mucus-like polymers play a key role in shaping the composition and structure of PGPB community (Broeckling et al., 2008; Carvalhais et al., 2015; Berendsen et al., 2018; Sasse et al., 2018; Yuan et al., 2018; Wen et al., 2020, 2021; Kong et al., 2021).

For example, long-chain fatty acids and amino acids were identified to play a crucial role in attracting PGPB, including *Pseudomonas* populations (Yuan et al., 2018; Wen et al., 2021). Additionally, a higher release of four short-chain organic acids (citric acid, pyruvate acid, succinic acid, and fumarate) has been linked to the increased presence of PGPB such as *Comamonadaceae* spp (Wen et al., 2020). Root-secreted malic acid has also been linked to the attraction of *Bacillus* spp. to the rhizosphere (Rudrappa et al., 2008). Therefore, the particular ratios and makeup of root exudates significantly influence the PGPB composition (Badri et al., 2009; Zhou and Wu, 2012).

Secondary metabolites secreted by plant roots can also be detrimental for the growth of specific group of microbes in the rhizosphere (Bais et al., 2002; Zhang et al., 2011). Benzoxazinoids are exuded in relatively large quantities from cereal roots and can inhibit rhizosphere microbes (Berendsen et al., 2012). In maize (*Zea mays*), 2,4-dihydroxy- 7-methoxy-2H-1,4-benzoxazin-3(4H)-one (DIMBOA) is the main antimicrobial benzoxazinoid. In contrast, PGPB *P. putida* KT2440 was attracted and tolerant to DIMBOA (Berendsen et al., 2012; Neal et al., 2012). In the absence of DIMBOA, the colonization of roots by KT2440 strain was lower (Berendsen et al., 2012; Neal et al., 2012). Secondary metabolites have shown promising nematocidal activity. Notably, various metabolites synthetized by wild watermelon roots have been documented in literature for their effectiveness in controlling nematodes (Kantor et al., 2018).

Plants also produce compounds that stimulate or repress quorum-sensing (QS)-regulated responses in PGPB. These QS-interfering compounds enable the plant to manipulate gene expression in their PGPB communities (Berendsen et al., 2012). PGPB utilize QS to signal each other and regulate expression of certain genes by using diffusible N-acyl-homoserine lactones (AHLs) (Elasri et al., 2001; Berendsen et al., 2012). AHL-mediated regulation typically makes use of two proteins that resemble the LuxI and LuxR protein families. LuxI-like proteins are AHL synthases, whereas LuxR-like proteins function as receptors of AHL that can form complexes with AHL which in turn can affect gene expression of QS-target genes (Decho et al., 2011; Berendsen et al., 2012). For instance, seedling extracts and exudates of barrel clover (*Medicago truncatula*), pea (*Pisum sativum*), rice (*Oryza sativum*) and green algae (*Chlamydomonas reinhardtii*) had compounds that specifically stimulated or repressed responses in QS-reporter bacteria (Teplitski et al., 2000; Gao et al., 2003; Teplitski et al., 2004; Ferluga and Venturi, 2009). Some plant-associated PGPB have LuxR-like proteins that are stimulated by plant-derived signals, whereas they themselves do not produce AHLs (Ferluga and Venturi, 2009; Berendsen et al., 2012). Thus, plants recruit and shape the rhizosphere microbes through the composition of root exudates and secondary metabolites. These substances selectively attract or repel soil microbiota and play a role in controlling the expression of QS-regulated genes of soil microbiota.

## 4 Interplay between host and PGPB in RKN suppression

Root exudates are important in nematode attraction to plant roots and directly affect nematode interactions with PGPB by inducing changes in the surface of PPNs. PGPB interact with PPNs through the nematode surface coat (SC). SC is a glycoprotein layer secreted by the hypodermis, or by the excretory and nervous systems (Lin and McClure, 1996; Curtis et al., 2011). Receptors on nematode SC mediate the specific interaction with the lectine-like protein molecules on PGPB surface (Bird, 2004; Davies and Curtis, 2011). Studies showed that nematode SC exposed to different root exudates and secondary metabolites also undergoes modifications which influence PGPB attachments to RKN surface (Akhkha et al., 2002; Curtis, 2008; Singh et al., 2014; Liu et al., 2017). *Pasteuria penetrans* endospores attachment to J2 of RKN were variable in response to root exudates from different plant species (Singh et al., 2014; Liu et al., 2017). For instance, *M. incognita* J2 exposed to the root exudates showed greater *P. penetrans* endospores attachment (Singh et al., 2014). These results indicated that the influence of specific host root exudates on RKN-PGPB interactions in the soil favors RKN antagonistic microbes attachment (Topalović et al., 2020c).

J2-attached PGPB can also increase hosts’ resistance to RKN. PGPB attaching to J2 of *M. hapla* prior to J2 infection enhanced their detection by upregulating several pattern-triggered immunity (PTI)-responsive defense genes (Topalović et al., 2020a). Moreover, chemicals produced by *M. hapla* J2 with attached *Microbacterium* sp. K6 strain activated a greater reactive oxygen species (ROS) response in tomato roots. Such a greater increase in ROS was not detected for nematodes without the K6 strain. Besides, hundred-fold ROS response was observed in the leaves than the roots for J2 with attached *Microbacterium* sp. K6 strain (Topalović et al., 2020a, b, c). Therefore, J2-attached PGPB prior penetrating roots can activate ISR that inhibits RKN establishment.

Recent research findings suggest that the success of RKN root invasion is influenced by the root exudates and PGPB in the rhizosphere which determine whether the RKN surface molecule is recognized by plant roots or not (Topalović et al., 2020c). Thus, host plant root exudates components play a key role for the communications between plants and nematodes, and nematode-PGPB interaction by modulating components of the nematode SC (Topalović et al., 2020c). Based on the host range of the nematode and the PGPB composition, RKN may either bypass plant defense responses to infiltrate the roots or be antagonized within or outside the plant. Thus, plants are utterly dependent on PGPB during nematode invasion, which results in the proliferation of a certain group of PGPB community protecting the host (Hussain et al., 2018; Topalović et al., 2020c). This suggests that the dynamic tripartite phenomenon in soil leads to nematode suppression by microbially induced systemic resistance in plants (Topalović et al., 2020a).

## 5 Soil microbes could enhance RKNs parasitism

RKN juveniles, while actively searching for roots in the soil, are likely to encounter and attach to a functionally diverse array of soil microbes. This array includes both antagonistic and protective surface microbes (Topalović and Vestergård, 2021). The holobiont concept suggests that each macroorganism has developed a mutually beneficial relationship with specific microbiota that influences its health and survival. Additionally, it infers that the microbial moiety of a holobiont can undergo modifications in response to environmental stress (Bordenstein and Theis, 2015). Soil microbes can protect PPNs in soil by outcompeting nematode antagonists for attachment sites on the nematode’s surface, reducing nematode recognition, or by producing compounds that are toxic to nematode antagonists (Topalović and Vestergård, 2021).

RKN J2s may avoid antagonists by recruiting protective soil microbiota to their surface. A recent study revealed that J2-attached microbes’ compositions were different on actively moving J2 surface of *Meloidogyne hapla* and *M. incognita* in the presence of *Pseudomonas protegens* strain CHA0, a bacterial antagonist (Topalović et al., 2023). In the absence of *P. protegens* strain CHA0, bacterial genera such as *Delftia*, *Variovorax* and *Pseudomonas* attached on both active and inactive J2s but not on J2 treated with *P. protegens* strain CHA0. *P. protegens* CHA0 also activated proliferation of *Flavobacterium* spp. and *Cutibacterium* spp., and Methylophilaceae family within the Gammaproteobacteria, which might have protective role on active nematodes in *M. hapla* and *M. incognita*, respectively (Tsuru et al., 2021; Topalović et al., 2023). The presence of *P. protegens* CHA0 might also change the surrounding microbial community by reducing the prevalence of nematode antagonistic taxa such as Pseudomonads may be due to a release of secondary metabolites from *P. protegens* CHA0 (Topalović et al., 2023). Such antimicrobial compounds might play a role in reducing the abundance of nematode antagonists in the soil in the presence of RKN protective soil microbiota.

PGPB attachment to nematode surface can reduce the nematode recognition by plants during the infection process by masking the nematode receptors (Curtis, 2008; Mendy et al., 2017; Topalović et al., 2020c). RKN surface-attached microbes may also facilitate RKN establishment by helping in the creation of a feeding site and enhancing nutrition available for the nematodes (Cao et al., 2015). Community analysis of root-associated microbiomes in healthy and RKN-infected tomatoes showed that nematode infections were associated with variation and differentiation of the endophyte and rhizosphere bacterial populations in plant roots (Tian et al., 2015). Bacterial genera with N-fixing (*Sinorhizobium* spp. and *Devosia* spp.) and cellulose-degrading (Sphingomonadaceae) abilities were found associated with different life stages of *M. incognita* on tomato (Cao et al., 2015; Tian et al., 2015). As the plant does not recognize N-fixing bacteria as pathogens, their introduction may deter RKN recognition and immune responses against the RKN. In addition, detecting cellulose-degrading bacterial groups may suggest that the gall-enriched cellulose-degrading bacteria may help nematodes in feeding site formation (Tian et al., 2015; Yergaliyev et al., 2020). Overall, soil type, plant genotype, the specific interaction between soil microbiota and nematode surface, and the movement of J2 influence the composition of J2-attached microbial community (Adam et al., 2014; Elhady et al., 2017; Topalović et al., 2019; Elhady et al., 2021; Topalović et al., 2023).

## 6 Role of abiotic factors in host-RKN interactions

**Figure 2:**
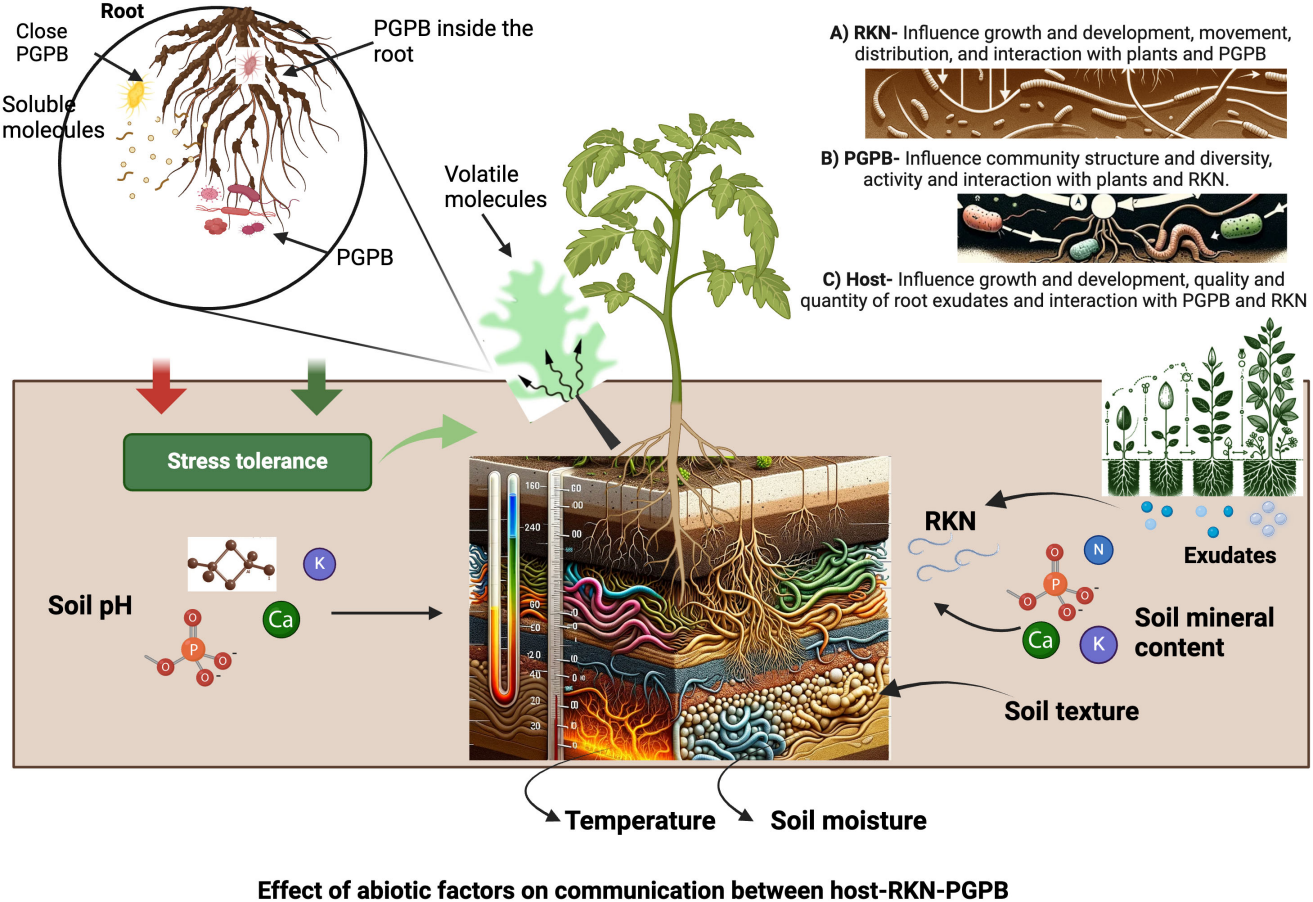
Impact of abiotic factors in host-RKN-PGPB interactions and their communications. The figure is created with BioRender.com.

Soil abiotic factors can affect host-RKN interactions through their impact on plant and RKN growth and development, and/or the activities of PGPB (**Figure 2**). RKN spend a phase of their life cycle (J2) in soil, the composition, and properties of which affect J2 motility and distribution, as well as their development inside their host (Norton, 1989; Mateille et al., 2014). Soil abiotic factors (soil physical properties such as temperature, texture, structure, and moisture content; soil chemical properties such as soil pH and mineral compositions) affect RKN behavior and development and in turn host-RKN interactions (Palomares-Rius et al., 2015). They also affect host growth and development such as root size, numbers, softness and quality and quantity of root exudates, and in turn RKN behavior and development (Castillo et al., 2006; Landa et al., 2014). Soil abiotic factors also affect the movement of volatiles released from roots and PGPB and alter the interactions of host-RKN-PGPB as volatiles play key roles in mediating intra- and inter-kingdom communications (Sharifi and Ryu, 2018; Erktan et al., 2020; Wester-Larsen et al, 2020; Lee and Ryu, 2021). While soil abiotic factors affect soil microbiota which in turn can affect host-RKN interactions as aforementioned, an in-depth analysis of this topic falls beyond the scope of this review as its main theme is to discuss the role of PGPB and abiotic factors on host-RKN interactions. Rather, in the following sections we will mainly discuss the major soil abiotic factors affecting host-RKN interactions by focusing on their impact on plants and RKN.

### 6.1 Soil temperature

Temperature influences nematode behavior such as egg hatching, nematode movement, root infection, their development and existence in soils (Velloso et al., 2022; Pradhan et al., 2023). Temperature also has a tremendous effect on plant development, reproduction, survival, and resistance to RKN (Hatfield and Prueger, 2015; Pradhan et al., 2023). Different levels of soil temperatures have variable effects on RKN root infection and their metabolism (Verdejo-Lucas et al., 2012, 2013; Khan et al., 2014; Pradhan et al., 2023). The tropical nematodes such as *M. incognita*, *M. javanica* and *M. arenaria* are most active for infection at a temperature of 24–32°C, while other root-knot nematodes such as *M. hapla* and *M. chitwoodi* can remain active in a temperature range of 10°C and 32°C (Davila-Negron and Dickson, 2013; Gine et al., 2021; Pradhan et al., 2023). As temperature increases, the number of RKN generations increase which leads to large increment in nematode population density and greatly reduce plant development (Verdejo-Lucas et al, 2013).

Generally, RKN reproduction increases when the soil temperature is intermittently above 28 °C (Talavera et al., 2009). However, temperatures below 18 °C decreased the J2 motility and subsequent root penetration and development inside roots (Roberts et al., 1981; Prot and Van Gundy, 1981a; Trudgill, 1995). High temperatures are also known to decrease nematode motility and cause lethality (Wallace and Bird, 1965; Wang and McSorley, 2008; Oka, 2019). Similarly, the pace of plant growth and development hinges on the ambient temperature of the plant, with each species having a defined temperature range represented by a minimum, maximum, and optimum temperature (Hatfield and Prueger, 2015). Soil temperature affects physiological processes of host plants such as root growth (Holtzmann, 1965), plant vigor and yield (Abdul-Baki, 1991), and thus, affect host-RKN interactions. For example, heat stress increase heat-shock proteins in plants that may alter the plant defense mechanisms at early stages of nematode infection (Verdejo-Lucas et al., 2013).

High temperature also affects plant resistance to RKN infection (Roberts, 2002). For instance, *Mi-1* gene is responsible for tomato resistance to *M. arenaria*, *M. incognita* and *M. javanica* (Smith, 1944) which greatly reduces the RKN reproduction in tomato (Roberts and Thomason, 1986; Sorribas et al., 2005). However, soil temperature above 28 °C usually negatively affects the resistance traits (Holtzmann, 1965; Dropkin, 1969; Araujo et al., 1982) and lead RKN to break the *Mi*-gene that lead to RKN population increase and affect plant growth and development (Devran and Sögüt, 2010; Verdejo-Lucas et al., 2012, 2013). As plants and RKN, PGPB have minimum, optimum, and maximum temperatures for their physiological activities. Temperature changes lead to structural and compositional changes in PGPR community which affect their interaction with plants and RKN, and host-RKN interactions (Zhang and Gross, 2021; Omae and Tsuda, 2022). The activity of PGPB enzymes can be influenced by soil temperature. For instance, the effectiveness of enzymes involved in nitrogen fixation varies at different temperatures (Abdul Rahman et al., 2021).

Temperature can influence the tripartite interactions through altering the host and PGPB volatiles concentration and mobility in soil (Kramshøj et al., 2019; Wester-Larsen et al, 2020). Studies showed that temperature increase positively correlated with an increase volatiles concentration by increasing biological activity, and liberating adsorbed and dissolved volatiles (Guenther et al., 1993; Insam and Seewald, 2010; Wester-Larsen et al., 2020). When concentration of volatiles in soil is low, it is not sensed over long distance by host, RKN and PGPB and hence affect the interactions between host-RKN-PGPB.

### 6.2 Soil texture, structure, and moisture content

Soil texture, structure and moisture are interrelated. Soil texture (proportions of sand, clay, and silt) and structure (soil aggregation) directly influence soil porosity which determines soil aeration, water infiltration and retention, and, indirectly, root growth, nematode movement, nutrient availability, and microbial activity (Saxton and Rawls, 2006; Mateille et al., 2014; Noronha et al., 2021; Garcia et al., 2022). Soil texture impacts the movements of J2 through the water film around soil particles, stimulated by the retention of root exudates that enable RKN to locate the roots (Prot and Van Gundy, 1981b; Garcia et al., 2022). The sandier soils seem to be good habitats for RKN and increase their presence in areas with coarse soil (Prot and Van Gundy, 1981b; Mateille et al., 2014; Kabir et al., 2017; Noronha et al., 2021). More structured soils with higher clay content, greater porosity, and water storage favored RKN because they retained water and created transport films in the soil that facilitated nematode movement (Otobe et al., 2004; Fajardo et al., 2011; Noronha et al., 2021). More structured soil also promotes the abundance, structure, and activity of PGPB which affects host plants, RKN as well as their interactions (Hartmann and Six, 2023).

Conversely, dry soil conditions may inhibit root growth, decrease metabolic activity, and cause electrolyte disturbances. These adverse effects can lead to the death of the plant, which in turn negatively affects the RKN development (Hurd, 1968; Roberts et al., 1981; Chen et al., 2022). Dry soils may not have enough water film for RKNs’ movement to locate host roots (Wallace, 1958; Oka, 2019). Dry soil also negatively affects the abundance, structure, and activity of PGPR and as a result of reduced nutrient availability, antipathogenic activities against RKN and the interactions between host and RKN (Bogati and Walczak, 2022; Omae and Tsuda, 2022). For instance, low soil moisture content decreases the movement of nitrogen-fixing bacteria to the rhizosphere, decreases rhizosphere colonization and their plant growth-promoting activity (Islam et al., 2020). Conducive soil physical properties such as water retention, porosity, aeration, and soil temperature enhance plant development, favor RKN and PGPB activity, and increase nematode and PGPB reproduction (Franchine et al., 2018; Notonha et al., 2021).

Soil texture, structure and moisture content affect the diffusion rate of volatiles in soil and as a result modulate the interactions between host, RKN and PGPB (Aochi and Farmer, 2005; Asensio et al., 2008). Soil texture and structure determine the pore sizes (micro or macro) in the soil which in turn influences the movement of soil organisms and soil moisture content (Mateille et al., 2014; Noronha et al., 2021; Garcia et al., 2022). The level of soil moisture content in turn affects the rate of diffusion of volatiles in soil that alters the interactions between host-RKN-PGPB. For instance, the movement of volatiles in wet soil is much slower than in dry soil, influences volatile travel distance and magnitude and impact the sensing ability of soil organisms such as RKN (Moldrup et al., 2000). In contrast, volatiles diffusion in the drier soil is faster and travels longer (Tyc et al., 2015; Erktan et al., 2020). RKN locate and move towards the host root tip by using the concentration gradient of volatiles as cue (Rasmann et al., 2012). Although RKN can better sense roots due to faster diffusion of volatiles, it may not reach to the root due to movement restriction in drier soil condition. Thus, optimal soil pore size and moisture content allows the movement of soil organisms and diffusion of volatiles. Similarly, the movement of PGPB to the root is mediated by the volatiles from the host, and plant roots must sense PGPB volatiles to respond accordingly (Schmidt et al., 2015; Schulz-Bohm et al., 2015; Tyc et al., 2017; Erktan et al., 2020; Sharifi et al. 22).

### 6.3 Soil pH

Soil pH is one the most important soil abiotic factors influencing soil properties, nutrient availability and solubility, plant growth, and RKN activity (Gentili et al., 2018; Penn and Camberto, 2019; Nisa et al., 2021; Barrow and Hartemink, 2023; Dewangan et al., 2023). Nutrient levels in soil are linked to the concentration of hydrogen ions, reflected in the soil’s pH value. Changes in pH level can influence the availability of nutrients, affecting plant growth. The exact influence of pH fluctuations on the soil’s microbial populations is not fully understood though it is known that pH is a key factor in determining microbial community structure (Biswas et al., 2007; Msimbira and Smith, 2020). Although the influence of soil pH varies with the host and nematode species (Wallace, 1973; Jones, 1975), soil acidity is a major abiotic stress factor that limits plant and RKN development (Schaller, 1987; Foy et al., 1993; Baligar and Fageria, 1997). In soil pH <5, for instance, aluminum (Al) becomes toxic to root growth while the essential nutrients such as P, K, magnesium (Mg) and calcium (Ca) become less available for uptake and negatively affect plant growth. Prolonged exposure to Al subjects plants to considerable oxidative stress and harms the root systems, impairing their ability to absorb water and nutrients (Kochian et al., 2005; Chai and Schachtman, 2022). Similarly, alkaline soils often have a reduced availability of P, zinc (Zn), Fe, copper (Cu), Boron (B), and manganese (Mn), which results in stunted plant growth (Melakeberhan et al., 2004; Barrow and Hartemink, 2023). Soil pH higher than 5 was associated with an increase of RKN populations; and pH values of 5.9 and 4.6 favored more pre-adult and adult stages of *M. incognita* than pH 4.3 in soybean roots (Melakeberhan et al., 2004; Kesba and Al-Shalaby, 2008; Nisa et al., 2021). Similarly, soil pH ranging from 5.7-7.9 appears to positively impact the abundance of RKN on sugarcane (Garcia et al., 2022). Based on the plant and RKNs species, specific range of soil pH negatively affects plants and RKN development and their interactions. Although tolerance of PGPB to soil acidity or alkalinity differs, most PGPR prefer pH of 6-7 and a change in range of soil pH alters their composition and activity which also alters their impact on host and RKN. For instance, low soil pH decreased nitrogen-fixing bacteria diversity and the process of N-fixation (Smercina et al., 2019; Abdul Rahman et al., 2021). Microorganisms in soil must have the ability to perceive and adapt to changes in their environment, including shifts in pH, to successfully survive and establish themselves (Biswas et al., 2007).

### 6.4 Soil organic matter

Increased soil organic matter (SOM) is typically linked to increased water holding capacity, storage of plant nutrients and structure of soil, and heightened microbial activity, and better plant growth, influencing host-RKN interactions (Pimentel et al., 2005; Evanylo et al., 2008; Zasada et al., 2008; Natsheh and Mousa, 2014; Zhang et al., 2014, 2016). One scenario illustrating the influence of SOM on host-RKN interactions involves the promotion of plant growth as indicated by previous studies (Pimentel et al., 2005; Forge and Kempler, 2009). This growth elevates the carrying capacity of plants on which RKN feed (Bongers et al., 1997; Bongers and Bongers, 1998; Bongers and Ferris, 1999; Habteweld et al., 2020a) or enhances microbial activity such as nematode antagonists resulting in RKN suppression (Gine et al., 2016; Silva et al., 2022). SOM also alters the tripartite interactions directly by adsorbing the volatiles released by plants and PGPB directly which decrease their concentration in soil or by involving soil structure and pore formation as aforementioned indirectly (Wester-Larsen et al., 2020).

### 6.5 Soil nutrient content

The presence of nutrients in the soil has a direct or indirect impact on both plant growth and development as well as RKN densities through the development of host plants (Habteweld et al., 2018). Soil mineral content is an important abiotic factor for nematodes’ development as they modify their habitat, metabolism, or movement (Norton, 1989; Mateille et al., 2014; Palomares-Rius et al., 2015). For instance, N is one of the macronutrients essential for plant growth and development and increase nematode reproduction indirectly by enhancing root growth (Santana-Gomes et al., 2013; Lira et al., 2019). Studies showed that high N content in the soil was positively correlated with RKN population densities in sugarcane and tomatoes (Asif et al., 2015; Ngeno et al., 2019).

P promotes root growth which increases nutrients acquisition and overall plant development (Devi et al., 2012). P deficiency induces the exudation of phenolics such as caffeic and protocatechuic acid into the rhizosphere resulting in desorption of P by binding with P-containing minerals in soils to release P for plant uptake (Juszczuk et al., 2004; Hu et al., 2005; Weisskopf et al., 2006; Noronha et al., 2021; Chai and Schachtman, 2022). P also influences RKN through biochemical changes in plants such as the increase in plant oils, phenolics, peroxidases, and ammonia that reduce the reproduction of the nematodes (Noronha et al., 2021). The addition of P fertilizers inhibits hatching and causes J2 mortality of *M. javanica* and *M. incognita* (Habash and Al-Banna, 2011; Hemmati and Saeedizadeh, 2019). K is required for plant development due to its involvement in various metabolic processes such as photosynthesis, protein synthesis, and translocation of sucrose from leaves to the stalk storage tissues (Medina et al., 2013). It is also related to stabilizing cell structure, thickening cell walls, and preventing the expansion of intracellular space (Li et al., 2010). Thus, low K levels in soil contribute to reducing the longevity of plants such as sugarcane (Noronha et al., 2021). K may suppress RKN as the application of K activates various enzymes improving plant resistance against *M. incognita* (Zhao et al., 2016).

While the impact on the development of RKN is not well studied, Ca, Mg, Ca/Mg, Carbon/Nitrogen (C/N) and soil cation exchange capacity (CEC) play important roles in the development of both plants and RKN. Ca is required for plant growth and development due to its involvement in cell wall and cell membrane formation, and N metabolism in plants (Thangavelu and Rao, 2004; Hepler, 2005). Mg is also required for plant growth and development due to its key role in photosynthesis and phosphorus transport (Thangavelu and Rao, 2004; Huber and Jones, 2013). A more recent study showed that increasing Ca/Mg ratio was associated with a decrease in RKNs’ densities (Noronha et al., 2021). C/N ratio and CEC improve soil nutrient retention capacity, enabling a steadier release of nutrients, thus having a positive impact on host and RKN populations (Garcia et al., 2022). The presence of heavy metals such as Zn or Cu in the soil suppresses RKN development (Park et al., 2011; Garcia et al., 2022). This effect could be indirect through reduced plant growth and thus lower quality nutritional content for RKN, as these organisms depend on their host plants for nutrition (Garcia et al., 2022). Thus, the imbalance of nutrients in the soil can affect the metabolism of the crop which can indirectly influence RKNs’ development (Coyne et al., 2004; Noronha et al., 2021; Garcia et al., 2022). Soil nutrients and their bioavailability influence the abundance, richness, and diversity of PGPB, and the interaction between host and RKN. For instance, addition of Fe and N influences microbial richness in the soil (Lakshmanan et al., 2014; Yang et al., 2015). Soil nutrients also influence the tripartite interaction by reducing the volatiles in soil. Sorption of volatiles to minerals are subject to degradation and catalyzed by mineral surfaces which reduce their diffusion and the sensing by plants, RKN and PGPB (Erktan et al., 2020).

## 7 Importance of agricultural practices in modulating host-RKN-PGPB interactions

Agricultural practices (APs) modulate the host-RKN-PGPB interactions by affecting plant and RKN development as well as altering soil’s physicochemical and biological properties (Habteweld et al., 2022). Common APs in conventional agriculture such as tillage, the use of inorganic fertilizers, and chemical pesticides and herbicides, may increase plant growth but often have harmful effects on the environment and human health (Lal, 2008; Diacono and Montemurro, 2010). For instance, conventional tillage has a negative impact on PPN populations by changing the physicochemical properties of the soil (Dick, 1992; Freckman and Ettema, 1993; Pankaj et al., 2006; Duplay et al., 2014). These changes can modify nematodes’ metabolism and reduce their mobility or access to food sources by removing weeds and altering their living habitats (e.g. living depth and soil structure) (McSorley and Dickson, 1990; Ou et al., 2005; Ekschmitt and Korthals, 2006; Mateille et al., 2014; Palomares-Rius et al., 2015; Garcia et al., 2022). The repeated use of synthetic fertilizers causes decline in soil physicochemical and biological properties (Odunze et al., 2012; Eche et al., 2013; Singh et al., 2013) that can in turn affect plant-RKN interactions. Acidic soil pH caused by the repeated application of chemical fertilizers negatively affects soil biological property which favors some pathogens. It also reduces plant growth, nutrient availability, and may affect the tripartite interactions (Singh et al., 2013; Habteweld et al., 2020a). N fertilizers, for example, promote plant growth leading to high carrying capacity for RKNs (Noronha et al., 2021) or decreasing RKN population density due to the release of nitrogenous compounds such as NH_3_ (Karajeh and Al-Nasir, 2012; Wei et al., 2012; Patil et al., 2013; 2014). Moreover, the use of insecticides and fungicides and soil disturbances due to tillage could eliminate potential natural enemies of RKN such as nematophagous fungi (Stirling, 2014; Kumar et al., 2017) leading to increased RKN populations and reduced plant growth (Garcia et al., 2022).

In contrast, cultural APs such as organic amendments, mulching, crop rotation, cover cropping and conservation tillage increase the availability of nutrients, improve soil structure leading to better moisture retention and soil microbial activity, reduce fertilizer loses to the environment, and increase plant growth (Oquist et al., 2007; Forge and Kempler, 2009; Glover et al., 2010; Zhang et al., 2014, 2016; Habteweld et al., 2020a, 2020b). Compost stands as one of the most widely employed organic amendments, demonstrating its ability to enhance soil organic matter, augment nutrient content, stimulate microbial activity, suppress pests, and contribute to overall soil health improvement (Bulluck et al., 2002; Forge and Kempler, 2009; Ferris et al., 2012; Habteweld et al., 2018, 2022). Incorporating compost as soil amendment increases soil pH by forming an aluminum complex and increasing base saturation (Shiralipour et al., 1992; Van den Berghe and Hue, 1999). In addition, organic amendments (composts, plant residues, animal manures, and plant derivatives) increase plant growth parameters (shoot fresh weight or dry weight) and decrease RKNs damage attributes, i.e. soil RKNs numbers, number of root galls, and number of eggs/egg masses in roots (Peiris et al., 2020). Organic APs are also known to enhance soil microbial activities including RKN antagonists (Silva et al., 2022). Rhizosphere soil under organic cultivation recruit RKN antagonistic bacteria genera such as *Pseudomonas*, *Serratia*, *Bradyrhizobium*, *Burkholderia* and *Azospirillum* and fungal genera such as *Beauveria*, *Clonostachys*, *Metarhizium*, *Purpureocillium* and *Arthrobotrys* (Silva et al., 2022). Thus, organic APs are potential candidates to modify soil and crop management as part of integrated strategies, thus enhancing the tripartite interactions towards RKN suppression and promoting plant growth and environmental safety.

## 8 Concluding remarks

RKNs are the most widespread PPNs in agricultural soils, infecting thousands of crops and causing annual losses of billions of dollars around the globe. The currently most effective and widely used RKNs control technique is the use of chemical nematicides. However, due to human health and environmental concerns, the use of many of these nematicides was banned or restricted. Therefore, there is a pressing need for effective and environmentally friendly alternative RKN control strategies. One such alternative is the use of RKN antagonistic microorganisms. However, microbial agents that were found to be effective in controlling RKN in the laboratory and/or in the greenhouse conditions often do not replicate the same level of control in the more complex soil ecosystems. The low efficacy of microbial agents may be attributed to overlooking native microbiota that possesses protective abilities for RKN, as well as to soil abiotic factors that modulate the host-RKN-PGPB interactions. Consequently, a deeper understanding of the dynamics of host-RKN interactions in varied biotic and abiotic environments could be pivotal in devising novel RKN control strategies.

## 9 Future perspectives of host-RKN-PGPB interactions for RKN mitigation

The utilization of PGPB for controlling RKN and fertilizing plants holds significant importance in agroecosystems, primarily due to their positive environmental impact. The application of PGPB, which facilitates RKNs’ control and increases soil fertility, plant growth, and crop safety, is poised to drive sustainable agriculture. However, the use of PGPB as an RKN control strategy requires a comprehensive understanding of the host-RKN-PGPB interactions and of how soil physicochemical and biological properties modulate the interactions. The concept of the holobiont indicates that plants have fostered a symbiotic relationship with specific microorganisms that play a role in their fitness, and that the microbial moiety of a holobiont can experience alterations in response to environmental stress (Bordenstein and Theis, 2015). Soil microbiota can also protect RKN in soil by outcompeting nematode antagonists for attachment sites on nematode surface, reducing nematode recognition, or by producing compounds that are toxic to nematode antagonists (Topalović and Vestergård, 2021; Topalović et al., 2023). So far, there are very limited studies to understand the role of RKN protective soil microbiota, soil edaphic factors and different agricultural practices in modulating the tripartite interactions. Hence, unraveling the tripartite interactions and understanding their relationship with soil biotic and abiotic factors may provide us with more knowledge on how to enhance PGPB efficiency in controlling RKN in agroecosystems. This knowledge may pave the way for the development of novel PGPB strains capable of competing and establishing themselves in soil ecosystems. It may also aid in selecting appropriate APs that increase PGPB efficiency. Moreover, the incorporation of PGPB into integrated RKN management strategies, particularly through APs such as organic amendments, cover cropping, and crop rotations, can improve soil physicochemical and biological properties. This, in turn, positively influences tripartite interactions, leading to more effective RKN control. However, several pressing questions remain to be addressed. For instance, how do we get deeper insight into the tripartite interactions to weaponize it for sustainable RKN management? How to find the most effective RKN-PGPB species combination that enhances host fitness? How does PGPB and RKN-protective microbiota competition influence the microbial composition in rhizosphere? What are the mechanisms RKN use to recruit protective soil microbiota in soil? What specific component of root exudates are involved in RKN and protective microbiota interactions? What abiotic factors favor RKN-protective microbes? Is RKN protective microbiota directly involved in infection and feeding site establishment? Which APs may help to enhance the abundance and activities of indigenous PGPB, and their communication through volatiles? Answers for these kinds of questions will lead to effective integration of PGPB in sustainable RKN control and ecologically sound agroecosystems.

## Author contributions

AH: Conceptualization, Writing – original draft. MK: Conceptualization, Writing – review & editing. CK: Conceptualization, Writing – review & editing. ZH: Conceptualization, Supervision, Writing – review & editing.
